# Verrue géante et récalcitrante: succès d'un traitement par photothérapie dynamique

**DOI:** 10.11604/pamj.2013.16.40.2438

**Published:** 2013-10-05

**Authors:** Mariame Meziane, Asmae Bettioui, Sanae Krich, Fatima-zahra Mernissi

**Affiliations:** 1 Service de Dermatologie, CHU Hassan II Fès, Maroc

**Keywords:** Verrue géante, photothérapie dynamique, acide methyl ester amino-levulinique, giant wart, PDT, acid methyl ester amino-levulinic

## Abstract

La photothérapie dynamique (PDT) utilisant l'acide methyl ester amino-levulinique est essentiellement utilisée dans les pathologies cutanées cancéreuses et précancéreuses. Son application au traitement des verrues est de description récente. Nous rapportons le cas d'un patient immunocompétent ayant une verrue géante et récalcitrante de la main droite traitée avec succès par la PDT, et discutons les difficultés de la prise en charge de ces verrues et l'intérêt de ce traitement dans l'obtention d'une bonne réponse thérapeutique et cosmétique avec peu de risque de récidive.

## Introduction

Les verrues constituent un motif fréquent de consultation en médecine générale et en dermatologie. Elles sont secondaires à une infection par les *Papillomas Virus Humains* (HPV). Ces lésions sont chroniques et peuvent disparaitre spontanément dans plus de 75% des cas au bout de 2 ans [1]; par ailleurs elles peuvent êtres responsables d'une altération de la qualité de vie, de gêne esthétique ou fonctionnelle. Leur traitement fait appel à de nombreux moyens thérapeutiques comme la cryothérapie et les agents kératolytiques qui constituent la base de la prise en charge. La photothérapie dynamique (PDT) est utilisée en dermatologie essentiellement en oncologie cutanée pour le traitement des kératoses actiniques, des carcinomes baso-cellulaires et de la maladie de Bowen. Récemment, plusieurs publications ont montré son effet bénéfique dans le traitement des verrues [2]. Nous rapportons le premier cas d'un patient immunocompétent ayant été traité pour une verrue géante et récalcitrante par la PDT avec d'excellents résultats.

## Patient et observation

**Figure 1:**
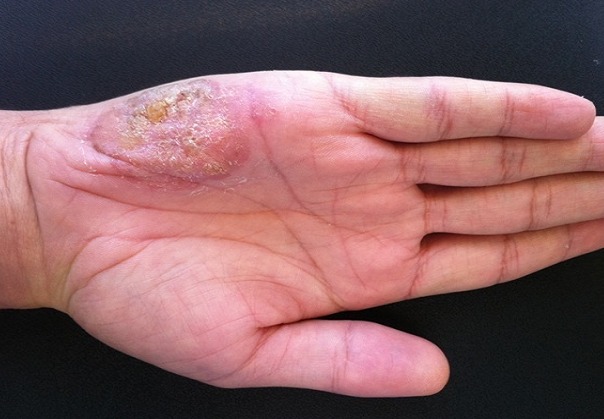
Aspect initial de la verrue géante avant le traitement

Un patient de 42 ans, sans antécédents pathologiques notables consultait pour une lésion verruqueuse au niveau de la main droite apparue depuis 8 ans. Il avait bénéficié de plusieurs traitements dont la cryothérapie, la vaseline salicylée et l'Imiquimod topique sans amélioration. L'examen retrouvait un sujet en bon état général, présentant un placard kératosique, rugueux à la palpation, mesurant 6x3cm et siégeant au niveau de l’éminence hypothénar de la main droite (Figure 1). Il n'existait pas d'autres lésions cutanées ou viscérales. Une biopsie cutanée a été réalisée; elle montrait un revêtement malpighien d'aspect verruqueux surmonté par une hyperkératose orthokératosique, sans signes d'atypie. Un bilan biologique (NFS, glycémie, sérologie VIH) était réalisé et était négatif. Le diagnostic d'une verrue géante récalcitrante a été retenu et le traitement par photothérapie dynamique (PDT) a été proposé.

Les lésions hyperkératosqiues ont été initialement traitées par de la vaseline salicylée à 20% pendant 15 jours, puis les lésions kératosiques résiduelles ont été curetées avant l'application d'une crème photosensibilisante à base d'acide méthyl ester amino-lévulinique (Metvixia^®^) sous occlusion pendant une durée de 3 heures. La source lumineuse était la PDT 1200 (Waldmann^®^, longueur d'onde 570-670 nm/cm^2^). Le patient avait reçu 2 traitements à 1 semaine d'intervalle avec une énergie de 75 Joules/cm^2^. Au cours de l'illumination, le patient avait rapporté une sensation de picotement et une douleur estimée à 7 sur 10 sur l’échelle analogique de la douleur qui a été par la suite réduite à 5 sur 10 après utilisation d'un système de refroidissement (cryoair) puis qui a disparue 3 heures après la fin du traitement.

**Figure 2:**
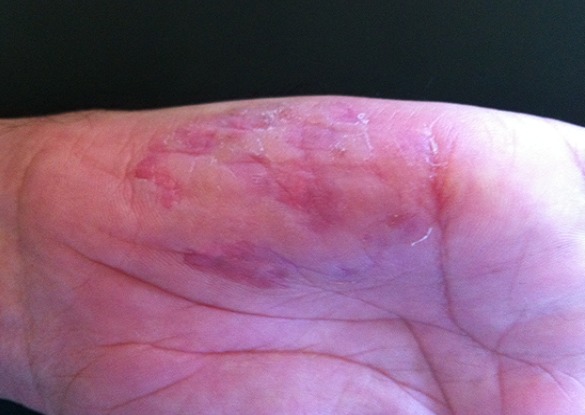
Résultat après la deuxième séance de PDT

**Figure 3:**
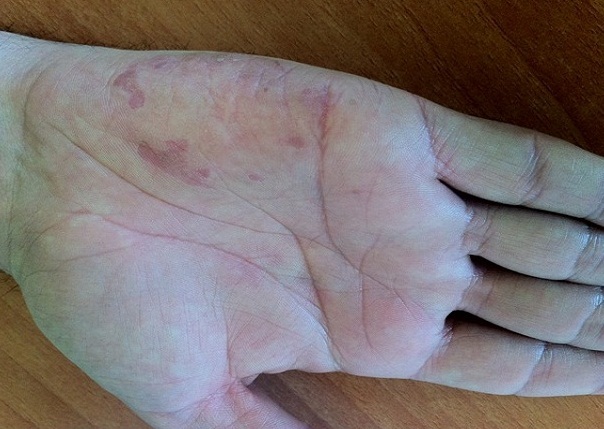
Résultat obtenu après 1 an de recul

Il n'existait pas de cicatrice, ou d'autres effets secondaires cutanés ou systémiques après traitement. Par ailleurs, on notait une amélioration très nette de la lésion de la main après 2 séances de PDT (Figure 2), sans récurrence après 1 an de recul (Figure 3).

## Discussion

Nous rapportons le premier cas d'une verrue géante et récalcitrante traitée par acide methyl ester amino-lévulinique-photothérapie dynamique (MAL-PDT) chez un patient immunocompétent.

Les verrues palmaires ou plantaires représentent 22% de l'ensemble des verrues cutanées vulgaires. Elles posent souvent un problème fonctionnel et esthétique [2]. Les verrues récalcitrantes sont des verrues qui résistent aux moyens thérapeutiques habituels (cryothérapie, vaseline salicylée..). Elles peuvent poser un problème de prise en charge. Plusieurs moyens thérapeutiques ont été décrits dans la littérature, ce qui témoigne de l'absence d'un seul moyen thérapeutique ayant une grande efficacité et peu d'effets secondaires [3]. La PDT constitue un des meilleurs moyens de prise en charge des verrues [4]. C'est un moyen thérapeutique sélectif reposant sur l'action conjointe d'une source lumineuse et d'un traitement photosensibilisant comme l'acide methyl ester amino-lévulinique (MAL) dont l'action est majorée par la réalisation d'un curetage des lésions avant le début du traitement. La PDT peut avoir un effet antiviral et un effet immunomodulateur. Son action antivirale pourrait être directe, grâce à une inactivation sélective des particules virales au niveau des kératinocytes infectés avec une nécrose cellulaire causée par une occlusion vasculaire des vaisseaux nourriciers [5] ou encore indirecte, suite à la destruction des cellules hôtes hébergeant le virus notamment les kératinocytes [2]. Par ailleurs, l'action immunomodulatrice consiste en l'amplification de la réponse immune anti-virale chez l'hôte, ce qui pourrait aider à éliminer les cellules infectées par le virus HPV et donc à diminuer le risque de récurrence suite à la persistance du virus [2].

Il n'existe pas de protocole standardisé pour le traitement des verrues récalcitrantes par PDT. En effet, la majorité des études ont été réalisées avec autre médicament photosensibilisant qui est l'acide amino-lévulinique avec des résultats intéressants: rémission complète chez plus de 75% des patients [6]. Par ailleurs, un seul cas a été retrouvé dans la littérature qui avait été traité par la PDT avec le MAL avec d'excellents résultats [7]. Il s'agissait d'un patient greffé rénal, sous traitement immunosuppresseur, et qui a été traité par MAL PDT à raison de 2 séances à 2 mois d'intervalle avec disparition totale et absence de récurrence après 5 mois de suivi [7]. Notre patient a été aussi traité par MAL-PDT, mais à raison de 2 séances à 1 semaine d'intervalle avec un très bon résultat.

Le principal effet secondaire rapporté au cours du traitement est la douleur; elle est décrite comme une sensation de brûlure, de picotement ou de fourmillement au niveau de la zone traitée pendant l'illumination. Le mécanisme de cette douleur est inconnu; mais il parait être la conséquence d'une stimulation nerveuse, ou de dommages nerveux secondaires aux espèces réactives d'oxygène générées au cours de l'illumination [8]. Cette dernière pourrait être majorée par les effractions cutanées secondaires au curetage manuel des lésions verruqueuses avant l'application du médicament photosensibilisant. L'intérêt de la PDT dans le traitement des verrues ne se limite pas aux lésions cutanées secondaires aux HPV mais aussi aux verrues génitales ou condylomes. Les résultats sont intéressants avec une meilleure réponse cosmétique par rapport aux thérapeutiques conventionnelles avec moins de risque de récidive [9].

## Conclusion

La PDT associée au MAL est une technique non invasive, avec peu d'effets secondaires et un bon résultat cosmétique. Elle peut être proposée comme une alternative thérapeutique intéressante dans la prise en charge des verrues géantes et récalcitrantes.
